# The Diagnosis of Smallpox

**Published:** 1912-06-08

**Authors:** 


### THE MAKING OF THE MODERN PRACTITIONER.

### The Diagnosis of Smallpox.

Some very useful practical points on the infective fevers are epitomised by Dr. Meredith Young in the *Practitioner*. On the importance of exact diagnosis in respect of smallpox he especially insists, because a single failure may involve an epidemic and a serious cost in life and money. If an eruption persists without appreciable alteration for three or four days, it is hardly ever variolous. Thus, peliosis rheumatica, lichen, and pustular syphilides can usually be excluded. On the other hand, the sudden onset of headache, slight pyrexia, and pain in the loins or sacrum, lasting for two days and disappearing when an eruption, *however atypical or slight*, appears, may safely be put down to smallpox; the eruption may consist of only a single spot, yet the case may be one of variola. It is the exception rather than the rule for severe prodromal symptoms to be followed by a copious eruption. Again, after suspicious symptoms of invasion, the appearance of an eruption upon the pharynx, palate, tongue, or conjunctiva, as well as upon the body, usually points to smallpox.

The most important characteristic of chicken-pox is the unilocular vesicle, which empties completely when pricked with a needle. Next in importance is the fact that fully-formed vesicles are visible within twenty-four hours (often earlier still) of the preliminary indisposition, which may be extremely slight. Successive crops of eruption are common in chicken-pox, so that papules, vesicles, pustules, and crusts may all co-exist. In smallpox, it is true, the rash appears earlier on the face and hands than on the body and lower limbs; but lesions in quite different stages of development are never seen. Umbilication of the vesicle, a feature of the true smallpox vesicle, is sometimes present in chicken-pox, but only when the vesicle has been ruptured or has arisen round a hair. In chicken-pox there is no alleviation of *malaise* when the rash comes out. The combination of lumbago with influenza may cause suspicions of smallpox; the only thing to be done is to await the eruption. The presence of pre-eruptive morbilliform, scarlatiniform, or hæmorrhagic rashes settles the point at once. These pre-eruptive rashes often give rise to mistakes and difficulties. The "triangular" rash of tiny petechiæ on a reddened skin, with the apex at the pubes and the base at the umbilicus, is absolutely characteristic of smallpox. Another pathognomonic rash is one formed of mingled large and small petechiæ filling Scarpa's triangle or, more rarely, a triangular space in the axilla. If either of these rashes is followed by bleeding from the nose, bowel, kidney, or uterus, the case is hæmorrhagic smallpox, and death will ensue about the third day.

After dealing with other sources of error, Dr. Young states that in rare cases a resort to vaccination may be the only means of clearing up the diagnosis. The physician should frankly avow his difficulty when proposing this test. A single negative result should not be regarded as final, if an eruption is actually present on the skin; several attempts should be made. Also a typical vaccinia pustule may not appear, in the same circumstances. The method is only applicable, naturally, to those previously unvaccinated.

